# Interaction of CD147 and human epididymis protein 4 promotes invasion and metastasis of ovarian cancer

**DOI:** 10.7150/jca.62440

**Published:** 2021-10-30

**Authors:** Lingling Gao, Xin Nie, Rui Gou, Yue Qi, Juanjuan Liu, Bei Lin

**Affiliations:** 1 Department of Obstetrics and Gynecology, Shengjing Hospital of China Medical University, No. 36, Sanhao Street, Heping District, Shenyang, 110004, China.; 2 Key Laboratory of Maternal-Fetal Medicine of Liaoning Province, Key Laboratory of Obstetrics and Gynecology of Higher Education of Liaoning Province, Liaoning, China.

**Keywords:** CD147, human epididymis protein 4, ovarian cancer, invasion and metastasis

## Abstract

**Background:** Ovarian cancer is one of the most common malignant tumors in female reproductive system. The expression of CD147 and human epididymis protein 4 (HE4) are both upregulated and associated with malignant progression in ovarian cancer. However, the important role of interaction between CD147 and HE4 in the invasion and metastasis of ovarian cancer remains unclear.

**Methods:** The co-expression and co-localization of CD147 and HE4 in cells and tissues of ovarian cancer were detected by co-immunoprecipitation, immunohistochemistry and immunocytochemistry, double-labeling immunofluorescence method. The interaction between CD147 and annexin A2 (ANXA2) was also investigated. Furthermore, we detect the regulatory relationship between CD147 and HE4 by Western blot. Transwell assay and scratch test were conducted to explore the effect of CD147-HE4 interaction on migration and invasion of ovarian cancer.

**Results:** The protein CD147 and HE4 were co-immunoprecipitated and co-located in the cytoplasm and membrane of ovarian cancer tissues and cells. Both of two proteins were highly expressed and positively associated with advanced FIGO stages, poor differentiation degree and poor prognosis of ovarian cancer, and HE4 was confirmed as an independent risk factor for CD147 in ovarian cancer tissues. What's more, AXNA2 was also identified as a CD147 interacting protein in ovarian cancer. It is further confirmed that interaction between CD147 and HE4 can affect the invasion and metastasis of ovarian cancer.

**Conclusion:** This study demonstrated that HE4 and ANXA2 were both CD147 interacting proteins, the expression of CD147 and HE4 can affect each other, and HE4 could promote the invasion and metastasis of ovarian cancer by regulating the expression of CD147, which may provide novel thought for early diagnosis and therapeutic target of ovarian cancer.

## Introduction

Ovarian cancer is one of the most common malignancy with the highest mortality rate in gynecological malignant tumors 1. Due to the concealment of location, lack of specific clinical features and early diagnostic methods, about 70% of the patients were in the advanced stages at diagnosis, ovarian cancer cells grew rapidly and were prone to invasion, migration and distant metastasis 2. Although the treatment strategy for ovarian cancer has been greatly improved in recent years, due to the metastasis, recurrence and drug resistance, the 5-year overall survival of patients with ovarian cancer was less than 47% 3. So far, many studies have showed varieties of novel biomarkers that can be treated as early diagnosis, prognosis evaluation and monitoring of recurrence of ovarian cancer. However, the development of reliable predictive molecules is urgently required to improve survival for ovarian cancer patients.

CD147, also known as extracellular matrix metalloproteinase inducer (EMMPRIN) is a single-stranded transmembrane glycoprotein, belonging to the immunoglobulin superfamily (IgSF) 4-5. The coding gene of *CD147* is located at chromosome 19pl3.3, which encodes a total of 268 amino acids (33-66kDa), including signal peptide, extracellular domain, transmembrane domain and intracellular region. The signal peptide region is involved in the biosynthesis and transport of CD147. There are two highly conserved Ig-like domains in the extracellular domain, the transmembrane domain can mediate the interaction between CD147 and membrane proteins. The protein exists in two forms: glycosylated form (HG~40-60 kDa) and core-glycosylated form (LG~32 kDa). CD147, due to different protein distributions and different glycosylation styles, could perform various functions in forms of high and low glycosylation in different tissues, while HG-CD147 is considered as the main functional form 6. Researches showed that CD147 could be involved in a variety of physiological and pathological processes, including spermatogenesis, embryonic development, tissue and organ formation, wound healing, inflammation, bone reconstruction and so on 7. At present, large number of studies have confirmed that CD147 was highly expressed in a variety of malignant tumors, and positively correlated with malignant progression of tumors, including breast cancer, hepatocellular carcinoma, gastric cancer, lung cancer, and ovarian cancer 8-12, which suggested that CD147 played a vital role in early diagnosis, prognosis evaluation, invasion and metastasis of various malignancy.

Human epididymal protein 4 (HE4), also called WAP four-disulfide core protein 2 (WFDC2), is an extracellular secretory protein containing two WAP domains, the *WFDC2* gene is located on chromosome 20q12-13 with a total length of about 12kb, consisting of 5 exons and 4 introns 13. Because of its specific localization in epididymal epithelial cells, HE4 was initially considered to be a biomarker of epididymal tissue 14. HE4 was proposed as a novel biomarker for ovarian cancer, and has been approved for clinical detection due to its high specificity and sensitivity in 2003^15^. In recent years, researchers have shown that HE4 was not only expressed in ovarian cancer, but also in other malignant tumors, such as endometrial carcinoma, lung cancer, breast cancer, gastric cancer and pancreatic cancer 16-19, which was closely correlated with the proliferation, invasion and migration of tumors.

We previously showed that CD147 and HE4 were both highly expressed in ovarian cancer tissues 11, and HE4 could interact with ANXA2 to promote malignant biological behavior and EMT process of ovarian cancer 16,20-21. Some studies have reported that there existed an interaction between CD147 and ANXA2 in hepatocellular carcinoma 22, but there's no evidence whether CD147 could interact with ANXA2 in ovarian cancer. Therefore, in this study, we will explore the interaction between CD147 and HE4 and their relationship with prognosis in ovarian cancer. The function of CD147-HE4 in invasion and metastasis of ovarian cancer was detected by cell function experiments, which will provide a new research direction to explore the interaction between CD147 and HE4 in ovarian cancer.

## Methods

### Patients and clinical tissue samples

A total of 133 ovarian tissues and paraffin-embedded specimens were obtained from Department of Obstetrics and Gynecology in Shengjing Hospital of China Medical University from 2004 to 2013. The pathological diagnosis of all samples was confirmed by in-house experts, as follows: malignant group, n = 96; borderline group, n = 11; benign group, n =14; normal group, n=12 (Table 1). All cases were primary epithelial ovarian cancer patients with complete clinical and pathological data, which were diagnosed by pathologists at the Shengjing Hospital of China Medical University, and patients with chemotherapy, radiotherapy, and hormone therapy before surgery were not included in this study (clinic-pathological parameters seen in Table 2). Patients in the malignant tumor group were 19-83 years of age (average: 55.21 years), those in the borderline tumor group were 26-84 years of age (average: 49.40 years), patients in the benign tumor group were 28-79 years of age (average: 55 years), and patients in the normal ovarian group were 36-57 (average: 44.17 years). No significant difference was found among the ages of each group (*P* > 0.05). The pathological types of ovarian cancer were as follows: serous adenocarcinoma (n=52), mucinous adenocarcinoma (n=7), endometrioid carcinoma (n=10), clear cell carcinoma (n=8) and other pathological types (adenocarcinoma) (n=19). In malignant ovarian tumor group, there were 11, 28, 57 cases of well, moderate, and poor differentiations, respectively. According to the criteria of the International Federation of Gynecology and Obstetrics (FIGO, 2009), each clinical surgical pathological stage was judged as follows: FIGO stages I-II (39 cases) and FIGO stages III-IV (57 cases). Lymph node metastasis was judged as follows: no metastasis (57 cases), metastasis (20 cases), and no lymphadenectomy (19 cases). This study was approved by the Ethics Committee of Shengjing hospital of China Medical University.

### Immunohistochemistry and Immunocytochemistry staining

Consecutive sections (5 μm thick) were processed from the ovarian tissue specimens fixed with 10% formalin and embedded in paraffin. The expressions of CD147 and HE4 in ovarian tissues were detected by streptavidin-peroxidase (SP) staining. Positive and negative controls were routinely used. The working concentrations of primary antibodies against CD147 and HE4 used were 1:150 (Proteintech, Wuhan, China, Cat# 11989-1-AP) and 1:50 (Abcam, Cambridge, UK, Cat# ab200828) respectively. The staining procedure was performed based on the manufacturer's instructions. The presence of buffy granules in the cell membrane and cytoplasm were regarded as positive. According to the chromatosis intensity, no pigmentation, light yellow, brown yellow, and dark brown are scored 0, 1, 2, and 3, respectively. The percentage of pigmented cells in the whole section was as follows: <5% % are 0, 5 % - 25 %: 1, 26 % - 50 %: 2, 51 % - 75 %: 3, and >75%: 4. The final score was obtained by multiplying the two scores above: 0-2 scores (-); 3-4 scores (+); 5-8 scores (++); and 9-12 scores (+++). Two pathologists who had no knowledge of the patient's data examined the sections independently to control error.

When cells grow at exponential phase, 0.25% trypsin were used to digest the cells, and mixed with culture medium containing 10% FBS to prepare single-cell suspension. When adherent cells grow in a single layer with 30-40% confluence, cells were washed with cold PBS three times, and then fixed with 4% paraformaldehyde for 20 min. The following procedures were conducted the same as immunohistochemistry. The working concentrations of primary antibodies against CD147 and HE4 were 1:100 (Proteintech, Wuhan, China, Cat# 11989-1-AP) and 1:50 (Abcam, Cambridge, UK, Cat# ab109298), respectively. PBS was used to replace the primary antibody for negative control, and secondary antibody alone was also used as a negative control.

### Cell culture and transfection

All ovarian cancer cell lines (CAOV3, SKOV3, OVCAR3, and ES-2) were obtained from the Institute of Biochemistry and Cell Biology, Chinese Academy of Sciences (Shanghai, China). CaoV3 and OVCAR3 cells were cultured routinely in RPMI 1640 culture medium with 10% fetal bovine serum (FBS). ES-2 and SKOV3 were propagated in McCoy's 5A with 10% FBS. All cell lines were grown at 37 °C in a humidified atmosphere with 5 % CO2.

OVCAR3 and ES-2 cells were employed to construct the stably low CD147 expression cells: OV-CD147-L group and negative control group (OV-NC) and ES-CD-147-L group and negative control group (ES-NC). The lentivirus-mediated CD147 RNAi sequence is: 5′-GUUCUUCGUGAGUUCCUCTT-3′. OVCAR3 and ES-2 cells were transfected with HE4 siRNA (GenePharma, Shanghai, China) using the Lipofectamine 3000 Transfection Kit (Invitrogen, USA) according to the manufacturer's instructions. HE4 siRNA sequences were as follows: siHE4-1: sense: 5′-ACCAGAACUGCACGCAAGATT-3′; antisense: 5′-UCUUGCGUGCAGUUCUGGUT-3′; siHE4-2: sense: 5′-AGGUGAACAUUAACUUUCTT-3′; antisense: 5′-GGAAAGUUAAUGUUCACCUTT-3′. The following cells of stably low ANXA2 expression were constructed: OV-shA2-1/OV-shA2-2 cells and negative control cells (OV-NC) and ES-shA2-1/ES-shA2-2 and negative control cells (ES-NC). The lentivirus-mediated ANXA2 shRNA (Hanbio Biotechnology, Shanghai, China) sequences are: GCTCTGTCATTGATTATGAACTGAT for ANXA2-shRNA1, TGGAGTGAAGAGGAAAGGAACTGAT for ANXA2-shRNA2. The interference effect was detected by Western blot.

### Double-labeling immunofluorescence

The double-labeling immunofluorescence was conducted to detect the co-expression and co-location of CD147 and HE4 proteins. Ovarian cancer tissues and cells were simultaneously incubated with primary antibodies against CD147 (1:100 [mouse], Santa Cruz, CA, USA, Cat# sc-21746) and HE4 (1:100 [rabbit], Abcam, Cambridge, UK, Cat# ab109298). The primary antibody was replaced by PBS for negative controls. The working concentrations of tetraethyl rhodamineisothiocyanate (TRITC) and fluorescein isothiocyanate (FITC) were 1:500. 4,6-diamidino-2-phenylindole (DAPI) was used to stain the nuclei. The following procedure was conducted according to the manufacturer's instructions.

### Co-immunoprecipitation and Western Blot

The pre-cooled RIPA buffer (500ul) was added to the precipitation of ovarian cancer cells OVCAR3 and ES-2, then incubated at 4 °C for 30 minutes. After centrifugated at 12000g for 30 minutes at 4 °C, the supernatant was collected and then added 2μg mouse anti-CD147 monoclonal antibody (Santa Cruz, CA, USA) and 2ug rabbit anti-HE4 monoclonal antibody (Abcam), and relative negative control mouse or rabbit IgG antibody (BIOSS, China) were added, respectively, followed by incubation overnight at 4 °C. 40 μL protein A/G-agarose bead (Santa Cruz, CA, USA) were added the next day and agitated at 4˚C for 4h. All samples were then centrifuged at 2,500 g for 5 min at 4˚C and washed with lysis buffer three times to collect the precipitation. The immunoprecipitation was analyzed by 10%SDS gel electrophoresis, and rabbit anti-HE4 monoclonal antibody (Abcam) and rabbit anti-CD147 polyclonal antibody (Proteintech) were used for western blot analysis. Proteins were detected with the Immobilon® western chemiluminescent horseradish peroxidase substrate (Millipore, Billerica, MA, USA). The experiments were repeated three times.

### Wound healing assay

Wound healing assay was conducted as the previous study, cells were seeded in a six-well plate. When cell confluence reached 90%, a 100-μL micropipette tip was applied to scratch a wound gently. The cells were washed three times with PBS and incubated with serum-free medium for 48h. The migration distance was observed under a microscope. The experiment was repeated three times.

### Transwell assay

Transwell assay was performed as the previous study. The upper Transwell chamber (Corning, Inc., Corning, NY, USA) was covered with 70uL basement membrane Matrigel solution (Corning, Bedford, MA, USA, Cat# 354234) and incubated overnight at 37 °C. A total of 2 × 10^4^ cells/200 μL were seeded in the upper chamber with serum-free medium, and the lower chamber was filled with 500 μL of medium supplemented with 10% FBS. After 48h of conventional incubation at 37 °C, the Transwell chambers were washed three times with PBS, then cells were fixed with 4% paraformaldehyde for 30 min and stained with crystal violet for 30 min, the Matrigel and cells from the inner surface of the chamber were gently removed with a cotton swab. The number of transmembrane cells was observed and counted under a microscope. The experiment was repeated three times.

### Statistical analysis

Statistical analyses were performed by SPSS21.0 software (IBM Corporation, Armonk, NY, USA), the data results were expressed as mean ± SD. Difference between two groups were calculated by student's t-test and the Chi-squared, and one-way ANOVA was used for comparison among more than two groups. The survival curve was measured by Kaplan-Meier and Log-rank test, and the correlation between the two proteins was analyzed by Spearman analysis and Regression model. Bilateral test *P* < 0.05 was regarded as statistically significant. *P<0.05, **P<0.01, and ***P<0.001.

## Results

### Co-expression of CD147 and HE4 in ovarian cancer cells and tissues

**Figure 1:**
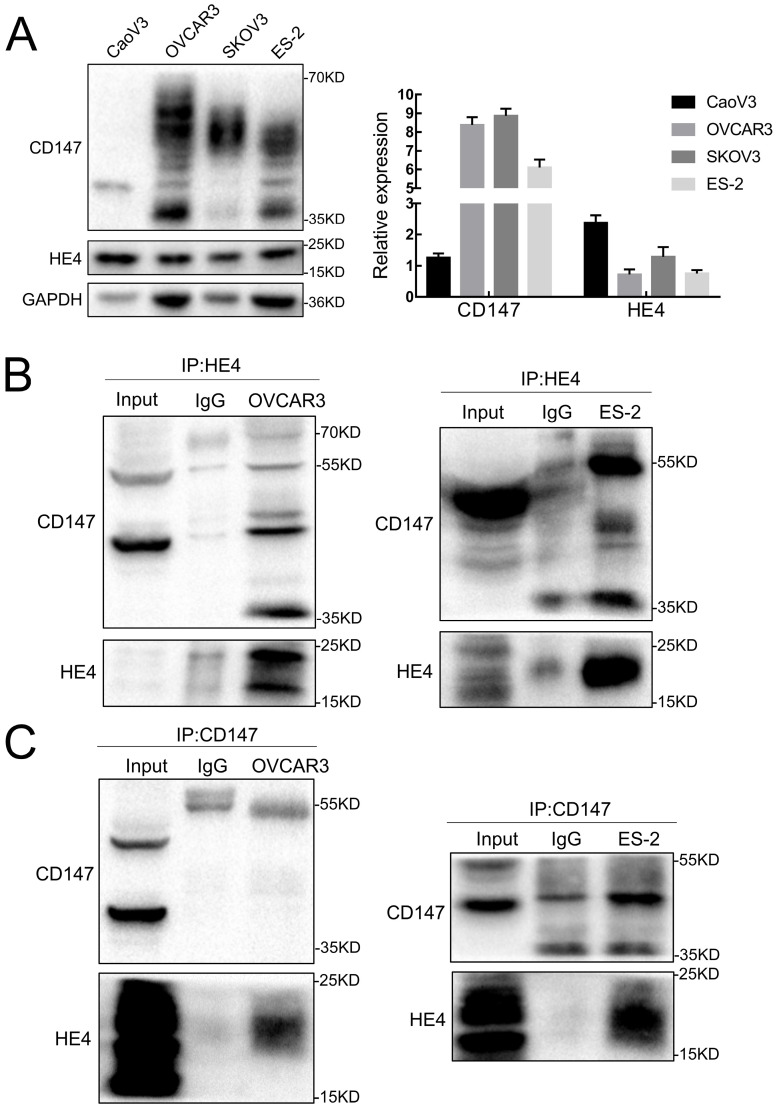
** Expression and co-immunoprecipitation of CD147 and HE4 in ovarian cancer cells. (A)** The expression of CD147 and HE4 in ovarian cancer cell lines (CaoV3, OVCAR3, SKOV3 and ES-2) detected by Western blot. **(B)** Cell lysates from OVCAR3 and ES-2 cells were subjected to immunoprecipitation with anti-HE4 antibody and immunoblotted with anti-CD147 antibody. **(C)** Cell lysates from OVCAR3 and ES-2 cells was immunoprecipitated with anti-CD147 antibody and immunoblotted with anti-HE4 antibody, “IgG” represents the negative control. “Input” indicates total cell lysate of OVCAR3 and ES-2.

**Figure 2:**
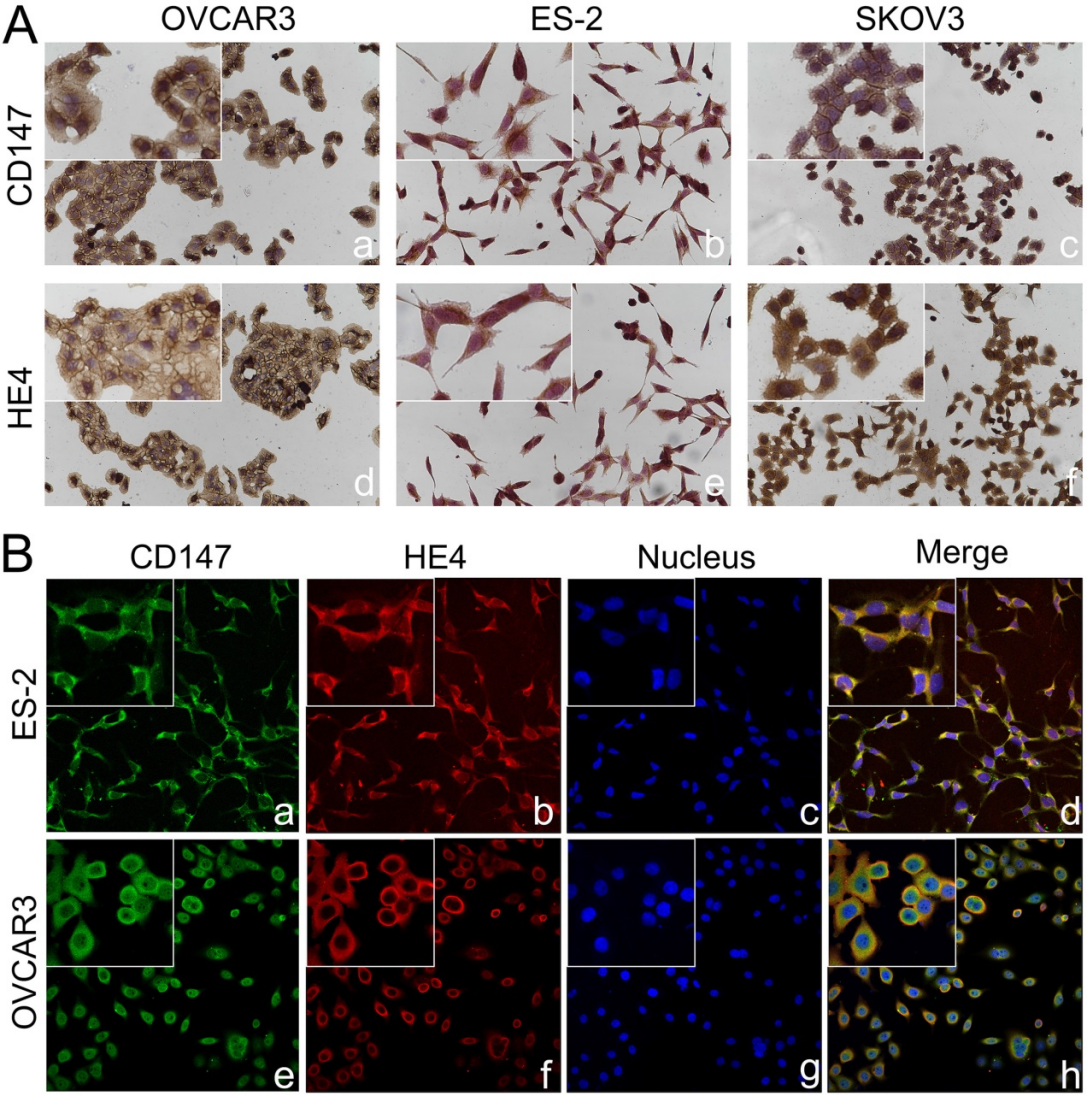
** Co-localization of CD147 and HE4 in ovarian cancer cells. (A)** Expression of CD147 and HE4 in ovarian cancer cell lines OVCAR3 (a, d), ES-2 (b, e), SKOV3 (c, f) conducted by immunocytochemistry. **(B)** The co-localization of CD147 (green) and HE4 (red) in OVCAR3 (a-d) and ES-2 cells (e-h) analyzed by double-labeling immunofluorescence assays, "blue" represents nucleus; "orange" represents the co-localization of CD147 and HE4. The small box at the top-left corner was magnified by 400× (A, B).

**Figure 3:**
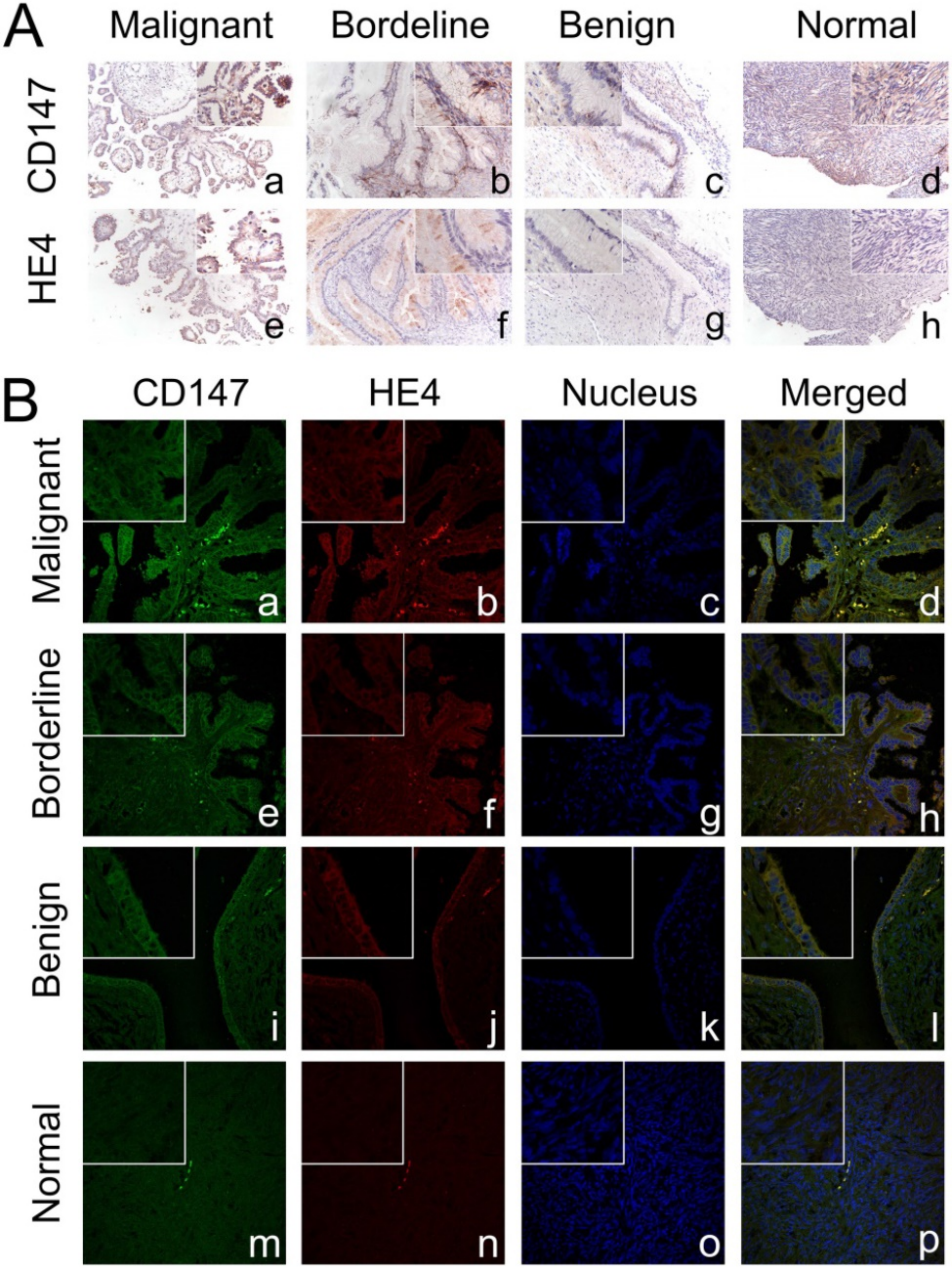
** Expression and co-localization of CD147 and HE4 in different ovarian tissues. (A)** The expression of CD147 and HE4 in malignant group (a, e), borderline group (b, f), benign group (c, g), and normal ovarian tissues (d, h) detected by immunohistochemistry. **(B)** Co-localization of CD147 and HE4 analysed by double-labeling immunofluorescence assay in malignant group (a-d), borderline group (e-h), benign group (i-l), and normal tissues (m-p). The color of “green” indicates CD147; “red” indicates HE4; “blue” indicates nucleus; “orange” indicates the co-localization of CD147 and HE4. The small box at the top-left corner was magnified by 400× (A, B).

The expressions of CD147 and HE4 protein in ovarian cancer cell lines OVCAR3 and ES-2 were detected by Western blot, immunocytochemistry and double-label immunofluorescence assays (Figure 1A and Figure 2A). We further confirmed the expression of CD147 and HE4 protein in different ovarian tissues with immunohistochemistry (Figure 3A). Interaction between CD147 and HE4 was detected by co-immunoprecipitation in OVCAR3 and ES-2 cells (Figure 1B-C). Double-label immunofluorescence assays showed that CD147 protein labeled by green fluorescence and HE4 protein labeled by red fluorescence were observed in the cell membrane and cytoplasm of OVCAR3 and ES-2 cells (Figure 2B) and ovarian cancer tissues (Figure 3B), and the co-localization sites of CD147 and HE4 were observed by the overlapping orange fluorescence. In addition, we also detected that CD147 can also interacted with ANXA2 in both OVCAR3 and ES-2 cells (Supplementary Figure 1A-B).

### Expression and co-localization of CD147 and HE4 in different ovarian tissues

Immunohistochemistry showed that CD147 was mainly expressed in the cell membrane and cytoplasm. The positive expression rate of CD147 in malignant group was 90.6% (87/96), which was significantly higher than that in borderline group (63.6%, [7/11]), benign group (42.9%, [6/14]), and normal ovarian tissues (33.3%, [4/12]) (all *P*<0.05). What's more, the positive expression rates of CD147 in borderline group and benign group were also higher than that in normal tissues, but the difference was not statistically significant (both *P*>0.05). According to the expression of CD147, 96 cases of ovarian cancer were divided into low (-/+) and high (++/+++) CD147 expression group, the results suggested that the high positive expression rate of CD147 in malignant group was 58.3% (56/96), which was higher than that in benign group (14.3%, [2/14]) and normal tissues (8.3%, [1/12]) (both *P* < 0. 05) (Table 1, Figure 3A).

HE4 was also mainly located in cell membrane and cytoplasm. The positive expression rate of HE4 in malignant group (86.5%, [83/96]) was higher than that in borderline group (54.6%, [6/11]), benign group (50%, [7/14]) and normal tissues (33.3%, [4/12]) (all *P*<0.05). Moreover, the positive expression rate of HE4 in borderline group and benign group was higher than that in normal tissues, but the difference was not statistically significant (both *P*>0. 05). We further divided malignant tumors into low (-/+) and high (++/+++) HE4 expression group, the results showed that the high positive expression rates of HE4 in malignant group, borderline group, benign group and normal group were 67.7% (65/96), 36.4% (4/11), 28.6% (4/14) and 16.7% (2/12), respectively. The high positive expression rate in malignant group was significantly higher than that in benign group and normal tissues (both *P* < 0.05) (Table 1, Figure 3A).

### Relationships between the expression of CD147, HE4 and clinicopathological parameters of ovarian cancer

**Figure 4:**
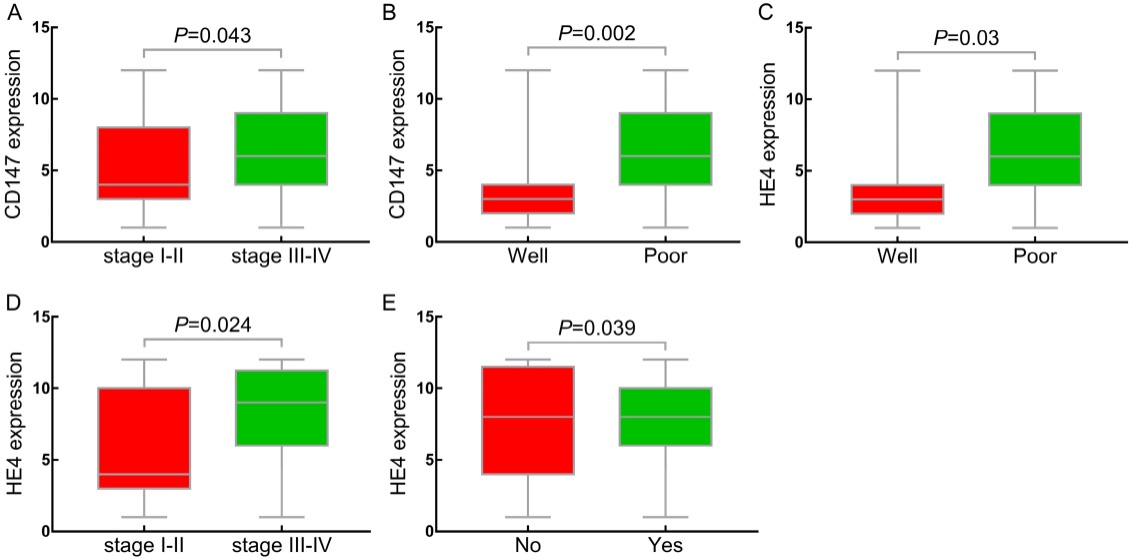
** Relationship between CD147, HE4 expression and clinicopathologic parameters. (A, D)** Compared the expression of CD147, HE4 protein in FIGO stages (I-II) with stages (III-IV) in OC, respectively; (B, C) Compared expression of CD147, HE4 protein in well differentiation group with poor differentiation group in OC, respectively. **(E)** Compared expression of HE4 protein in lymph node metastasis with non-metastasis group in OC. OC: ovarian cancer.

The positive expression rate of CD147 in stages III-IV of ovarian cancer was 96.5% (55/57), which was higher than that in stages I-II (82.1%, [32/39]) (*P<*0.05) (Table 2, Figure 4A). The positive expression rate of CD147 in the poor differentiation group (96.5%, [55/57]) was significantly higher than that in well differentiation group (63.6%, [7/11]) (*P<*0.05) (Table 2, Figure 4B). The positive expression rate of CD147 in lymphnode metastasis group was 95% (19/20), which was higher than that in non-metastasis group (87.7%, [50/57]), but no statistical significance (*P*>0.05). 96 cases of epithelial ovarian malignant tumors were further divided into low (-/+) and high (++/+++) CD147 expression group based on the CD147 expression in ovarian cancer. The results indicated that the high positive expression rate of CD147 in III-IV stages (66.7%, [38/57]) was higher than that in stages I-II (46.2%, [18/39]) (*P<*0.05). The high expression rate of CD147 gradually decreased as the degree of differentiation increased, and the high positive expression rate in low differentiation group (70.2% [40/57]) was significantly higher than that in well differentiated group (18.2%, [2/11]) (*P<*0.05). Although the high expression rate in the low differentiation group was also higher than that in the moderate differentiation group, the difference was not statistically significant. No significant difference in the expression of CD147 with respect to age at diagnosis and pathological type (*P*>0. 05) (Table 2).

In accordance with CD147, the positive expression rate of HE4 in III-IV stages (93%, [53/57]) was significantly higher than that in stages I-II (76.9%, [30/39]) (*P*<0.05) (Table 2, Figure 4C). The positive expression rate of HE4 in poor differentiation group was 91.2% (52/57), which was significantly higher than that in high differentiation group (63.6%, [7/11]) (*P*<0.05) (Table 2, Figure 4D). Similarly, we further divided the ovarian epithelial malignant tumor group into HE4 low (-/+) and high (++/+++) expression group. The results showed that the high positive expression rate of HE4 in stages III~IV (80.7%, [46/57]) was significantly higher than that in stages I ~ II (48.7%, [19/39]) (*P*<0. 05). With the decrease of differentiation degree, the expression rate of HE4 increased gradually, and high positive expression rate in low differentiation group (75.4%, [43/57]) was significantly higher than that in high differentiation group (27.3%, [3/11]) (*P*<0.05). The high expression rate of HE4 in lymph node metastasis group was 85% (17/19), which was significantly higher than that in non-metastasis group (59.6%, [34/57]) (*P*<0.05) (Figure 4E). No significant difference was showed in the expression of HE4 with age at diagnosis and pathological type (*P*>0.05) (Table 2).

### CD147 and HE4 overexpression associated with poor prognosis in ovarian cancer

**Figure 5:**
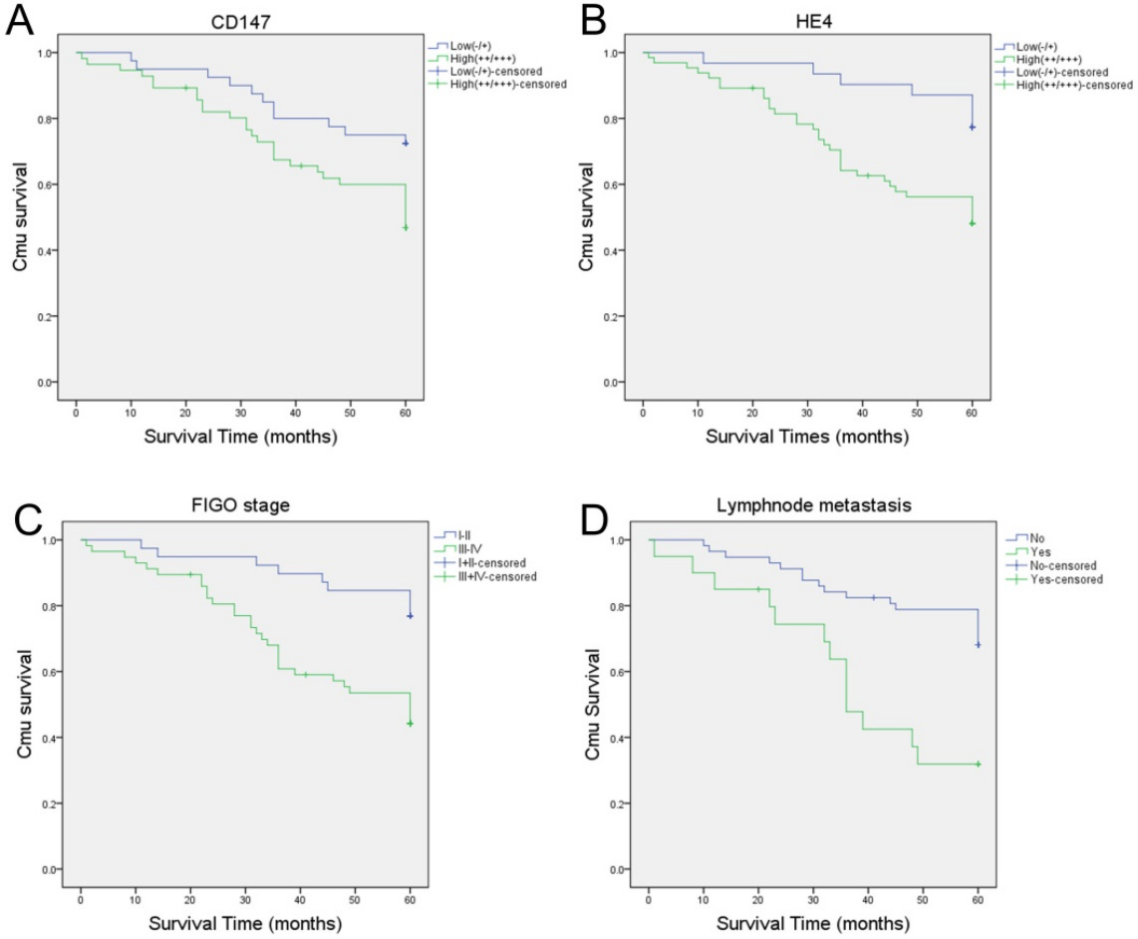
** Kaplan-Meier analysis of the prognosis of ovarian cancer.** Relationship between prognosis and high or low CD147 expression (**A**), high or low HE4 expression (**B**), FIGO stages (**C**), lymphnode metastasis (**D**).

A total of 96 cases of ovarian cancer patients were followed up until January 1, 2018. Among these ovarian cancer patients, 14 patients were lost follow-up and 40 died. The longest and shortest survival time were 119 and 1 months, respectively. The mortality rates of patients with high expression of CD147 and HE4 were 52.9% (29/56) and 50.8% (33/65) in epithelial ovarian malignant tumors, respectively, which were significantly higher than that in CD147 low expression group (27.5%, [11/40]) and HE4 low expression group (22.6%, [7/31]). Kaplan-Meier analysis showed that the average survival time in CD147 and HE4 high expression group were 45.73 and 44.92 months, respectively. While the average survival time in CD147 and HE4 low expression group were 52.65 and 56.35 months, respectively. The high expression of CD147 and HE4 was significantly associated with a shortened overall survival in patients with ovarian cancer (*P*=0.018 and *P*=0.005, respectively) (Figure 5A-B). Furthermore, FIGO stages (I-II vs III-IV) and lymph node metastasis (No vs Yes) were also associated with poor prognosis in ovarian cancer (all *P*<0. 05) (Supplementary Table 1, Figure 5C-D).

**Figure 6:**
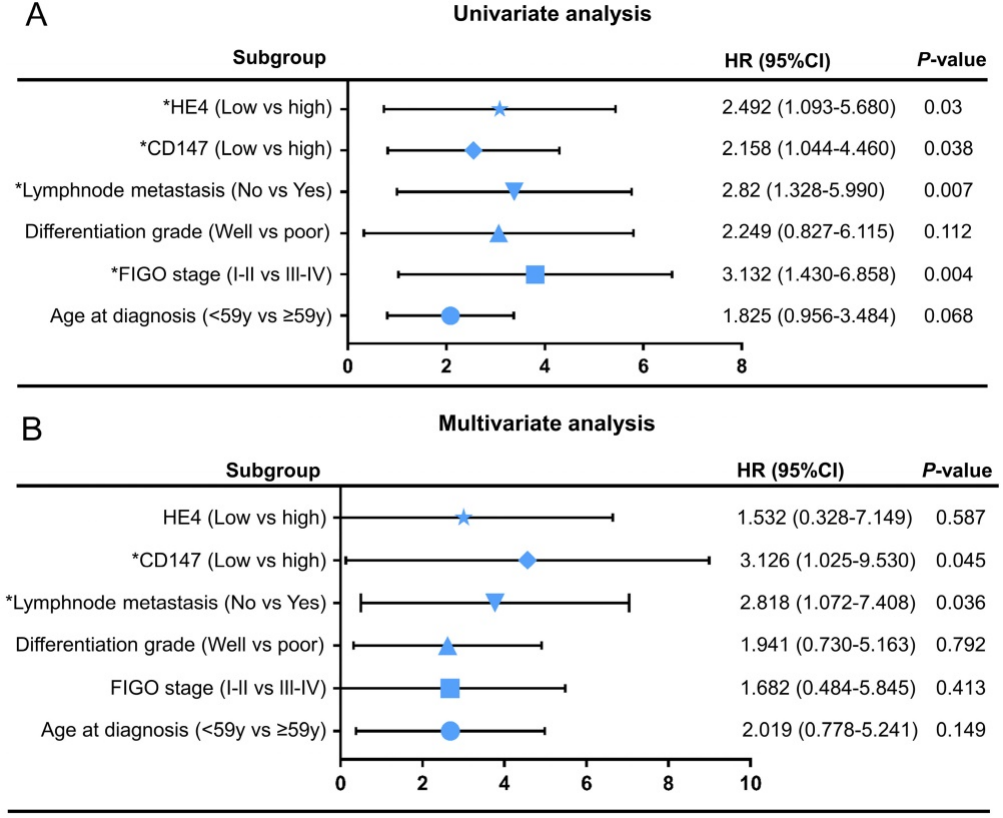
Univariate** (A)** and multivariate** (B)** Cox regression analyses by forest map. *P<0.05

Cox regression model was adopted to analyze the clinicopathological parameters affecting the prognosis of patients with ovarian cancer. Univariate analysis showed FIGO stages (HR=3.132, 95% CI=1.430-6.858,* P*=0.004), lymphnode metastasis (HR=2.82, 95% CI=1.328-5.990, *P*=0.007), CD147 expression level (HR=2.158, 95% CI=1.044-4.460, *P*=0.045) and HE4 expression level (HR=2.492, 95% CI=1.093-5.680, *P*=0.03) were significantly correlated with overall survival time (Table 4). Multivariate analysis showed that CD147 expression level (HR=3.126, 95% CI=1.025-9.530, *P*=0.004) and lymph node metastasis (HR=2.818, 95% CI=1.072-7.408, *P*=0.036) were independent risk factors affecting the prognosis of ovarian cancer. The univariate and multivariate Cox regression analysis were further visualized by forest maps (Figure 6).

### Correlation between the expression of CD147 and HE4 proteins in ovarian cancer tissues

According to the expression of CD147 and HE4 in 96 cases of ovarian cancer, there were 8, 1, 5, and 82 patients in the CD147-/HE4-, D147-/HE4+, CD147+/HE4- and CD147+/HE4+ groups, respectively. Spearman analysis and Regression model showed that the expression of CD147 protein was positively correlated with HE4 protein in ovarian cancer (Spearman correlation coefficient *Rs* = 0.708, *P* = 0.000) (Table 3, Supplementary Figure 2). Univariate linear regression indicated that the expression of CD147 and HE4 could interact with each other (both *P*<0 05). As shown in Table 4, the degree of differentiation can significantly affect the expression of CD147, and the advanced stages are important factors affecting the expression of HE4. Multivariate linear regression suggested that HE4 expression score (0-12) was an independent influence factor of CD147 expression, and CD147 expression was also an independent factor of HE4 expression (Table 4).

### Interaction between the expression of CD147 and HE4 in ovarian cancer cells

**Figure 7:**
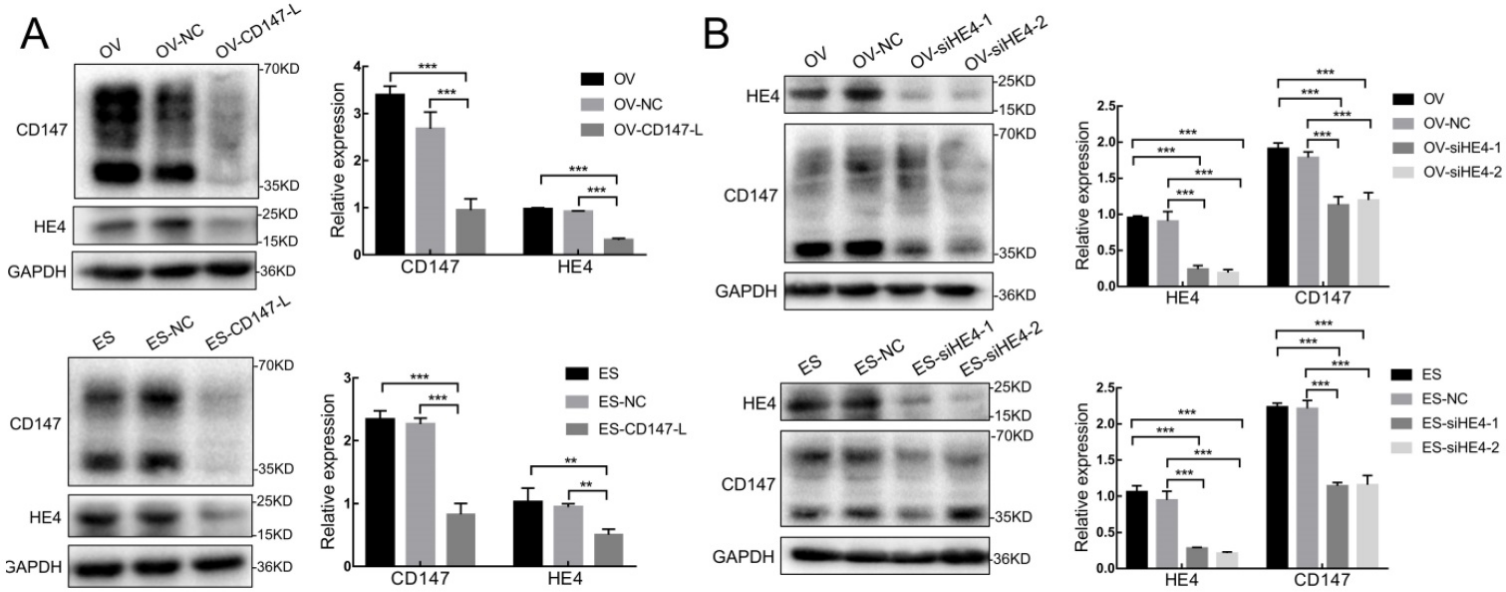
** Interaction between CD147 and HE4 expression. (A)** The expression of HE4 protein decreased after downregulation of CD147 protein in OVCAR3 and ES-2 cells by Western blot. **(B)** The expression of CD147 was downregulated after inhibition of HE4 protein in OVCAR3 and ES-2 cells by Western blot. OV: OVCAR3, ES: ES-2, HE4: human epididymis protein 4, OV-NC: negative control group of OVCAR3 cells, ES-NC: negative control of ES-2 cells, OV-CD147-L: downregulation of CD147 expression in OVCAR3 constructed by lentivirus, ES-2-CD147-L: downregulation of CD147 expression in ES-2 constructed by lentivirus, OV-siHE4-L-1/2: downregulation of HE4 expression in OVCAR3 by siRNA, ES-siHE4-L-1/2: inhibition of HE4 expression in ES-2 by siRNA.

We further constructed the stable low expression CD147 ovarian cancer cells OV-CD147-L and ES-CD147-L and their negative control groups OV-NC and ES-NC by lentivirus transfection, the results showed that the expression of HE4 decreased and the expression of AXNA2 protein did not change significantly after the expression of CD147 was inhibited in OV-CD147-L and ES-CD147-L cells by Western blot (Figure 7A and Supplementary figure 3A). Furthermore, the expression of HE4 was inhibited by gene transfection and RNA interference, the result indicated that the expression of CD147 protein in high and low glycosylation forms was decreased after inhibition of HE4 expression (Figure 7B). Stable low expression of ANXA2 ovarian cancer cells OV-shA2-1/OV-shA2-2 and ES-shA2-1/ES-shA2-2 and their negative control groups OV-NC and ES-NC were constructed by lentivirus, the results showed that after inhibition of ANXA2 expression, there was no significant change in the expression of CD147 protein (Supplementary Figure 3B). These results suggested that the expression of CD147 and HE4 could interact with each other, however, the expression of CD147 and ANXA2 had no significant effect on each other.

### Interaction between CD147 and HE4 promotes invasion and metastasis of ovarian cancer

**Figure 8:**
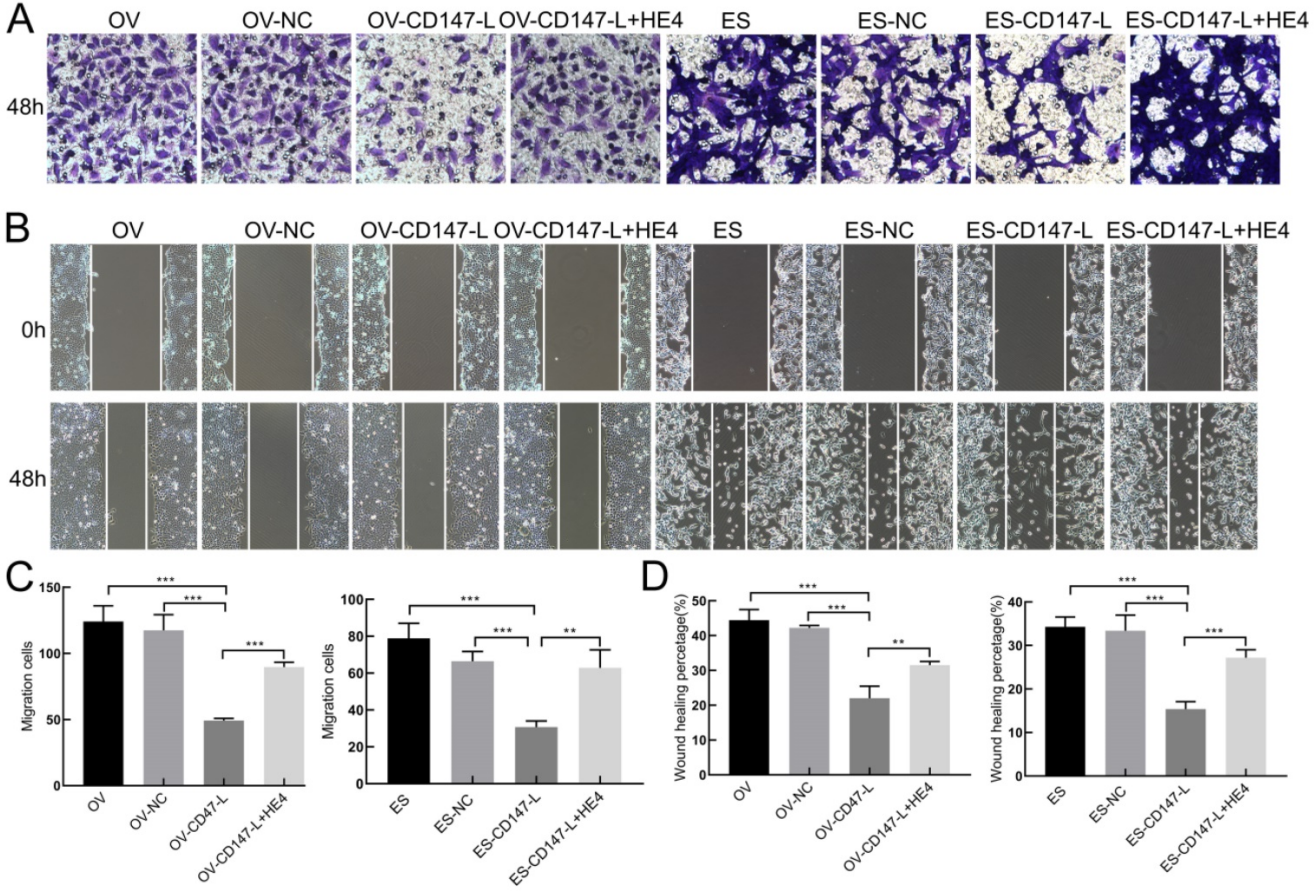
** Interaction of CD147 and HE4 promotes invasion and migration of ovarian cancer cells. (A, C)** The invasion capacities of ovarian cancer cells (OVCAR3 and ES-2) after downregulation of CD147 protein and addition of recombinant HE4 active protein detected by Transwell assay. **(B, D)** The migration capacities of ovarian cancer cells (OVCAR3 and ES-2) after downregulation of CD147 protein and addition of recombinant HE4 active protein detected by Scratch test. HE4: human epididymis protein 4.

In order to further detect the role of CD147 and HE4 interaction in the invasion and metastasis of ovarian cancer cells, Transwell assay and Scratch test were performed. The results showed that the invasive and migration abilities both decreased after downregulation of CD147 protein in OVACR3 and ES-2 cells, and recovered after the addition of human recombinant HE4 active protein by Transwell assay and Scratch test (Figure 8). These results suggest that HE4 may affect the invasion and migration of ovarian cancer cells by regulating the expression of CD147.

## Discussion

The progression and development of malignant tumor is a multi-factor, multi-step cascade reaction process, including infiltration of primary tumor, degradation of basement membrane, invasion of tumor cells and distant metastasis. Degradation of extracellular matrix is the premise of tumor cell invasion and metastasis, as an extracellular matrix metalloproteinase inducer, CD147 is a cancer-specific biomarker, which can not only mediate intercellular adhesion, but also induce production of extracellular MMPs, playing a key role in promoting tumor invasion and metastasis 23-24. Researchers found that CD147 protein was up-regulated in many kinds of tumors, such as endometrial carcinoma, colon cancer, liver cancer and lung cancer, regulating malignant progression, infiltration, and invasion of the tumors 25-26, 8, 12. Studies have shown that CD147 can regulate cytoskeletal movement by activating PI3K/Akt and MAPK signaling pathway, induce the production of vascular endothelial growth factor (VEGF), and enhance anoikis resistance 27-29. Several investigations demonstrated that CD147 can facilitate the glycolysis of tumor cells in anoxic microenvironment, enhance tumor proliferation, invasion, and inhibit apoptosis under hypoxic condition 30-31. At present, anti-CD147 monoclonal antibody can inhibit the invasion ability of liver cancer by reducing the expression of MMP2 and MMP9. What's more, anti-CD147 monoclonal antibody can inhibit the occurrence and development of pancreatic cancer, suggesting that anti-CD147 monoclonal antibody has potential clinical value in drug-resistant recurrence of pancreatic cancer 32.

As a new generation tumor marker of ovarian cancer, HE4 is an exocrine protein encoded by WFDC2 (WAP four-disulfide core protein 2) gene 33. At present, the research on HE4 is mainly focused on ovarian cancer and endometrial cancer, the level of *HE4* gene in ovarian serous carcinoma is significantly higher than that in other cancers, the levels of *HE4* gene expression are moderate in transitional cell carcinoma, breast cancer, renal cell carcinoma and pancreatic cancer. However, the expression of *HE4* gene was very low in colon cancer, gastric cancer, liver cancer and prostate cancer 34, which may be associated with different genetic backgrounds in various types of tumors. In 2013, Kong et al found that HE4 could inhibit the proliferation abilities of ovarian cancer cells through MAPK and PI3K/AKT signaling pathway, but had no effect on EGFR phosphorylation 35. Zhuang et al showed that Lewis y fucosylation of HE4 protein promoted the proliferation, invasion and metastasis of ovarian cancer cells 36. HE4 protein is also significantly increased in endometrial carcinoma, which is helpful for the early diagnosis and treatment of endometrial carcinoma 16. Furthermore, the high expression of HE4 is associated with lymph node metastasis and shortened disease-free survival in breast cancer, suggesting that HE4 can be used as a prognostic factor for breast cancer 37. The above studies have suggested that HE4 plays a vital role in the occurrence, development, and biological function of malignant tumors.

In order to investigate the expression and mutual interaction of CD147 and HE4 in ovarian cancer, we confirmed that these two proteins co-precipitated and co-located in ovarian cancer cells by Western blot, immunoprecipitation, and double-labeling immunofluorescence assays. We further confirmed that both CD147 and HE4 proteins were highly expressed in ovarian cancer tissues, and there was a positive correlation between the expression of CD147 and HE4 protein, suggesting that the interaction between CD147 and HE4 plays a crucial part in the occurrence and development of ovarian cancer. Furthermore, high expression of CD147 and HE4 was significantly associated with the differentiation degree and FIGO (III-IV) stages of ovarian cancer, and predicted poor prognosis of ovarian cancer, indicating that the interaction between the two molecules participated in promoting the invasion and metastasis of ovarian cancer and affecting the prognosis of patients with ovarian cancer. Taken together, this study may provide basis therapy for further exploring the interaction of CD147 and HE4 in other malignant tumors.

Most proteins usually form molecular complexes together performing their biological functions in the process of invasion and metastasis of malignant tumors. At present, numerous studies have shown that CD147 can interact with varieties of molecules to regulate the occurrence, development, invasion, and metastasis of tumor, including Annexin A2 (ANXA2), VEGFR-2, MCT1, integrin-β1, CD44, cyclophile. Zhao et al. 22 showed that CD147 interacted and co-precipitated with ANXA2 in hepatocellular carcinoma cells, and could promote the movement and invasion of tumor cells. CD147 also regulated the activation of VEGFR-2 and downstream signaling pathway by directly binding to VEGFR-2 38. Walters et al 39 showed the interaction of CD147 with MCT1 and MCT4 promoted glycolysis and accelerated the malignant progression of tumors. Additionally, the interaction between CD147 and integrin-β1 promoted the invasion and metastasis of hepatocellular carcinoma 40. In melanoma cells, CD147 interacted with cyclophile to regulate Ca^+^ channel and MMPs activities 41.

At present, studies showed that HE4 can interact with HIF-α, EGFR, IGF1R, ANXA2 and other protein molecules. Researchers found that the expression of HE4 decreased after inhibition of HIF1-α expression or treated with HIF1-α inhibitor in tumor cells; HE4 could co-immunoprecipitate and co-locate with EGFR, and the interaction between HE4 and EGFR was enhanced after upregulation of HE4 expression 42. Our study suggested that the invasion and migration abilities were both inhibited after downregulation of CD147 protein and recovered after the addition of human recombinant HE4 active protein, indicating that CD147 and HE4 could interacted with each other to form a protein complex, and HE4 could promote invasion and metastasis of ovarian cancer by regulating the expression of CD147. We further confirmed that ANXA2, as another CD147-interacting protein in ovarian cancer, had no effect on the expression of CD147. Previous studies have found that the interaction between ANXA2 and HE4 promotes the invasion and migration of ovarian cancer cells by activating MAPK and FOCAL signaling pathways 16,20. Moreover, HE4-AXNA2-MMP2 can form protein complex to promote the invasion and metastasis of malignancy 21. Therefore, we speculate that the interaction between CD147 and HE4 may regulate the occurrence, development, invasion and metastasis of ovarian cancer in the form of protein complex, in which ANXA2 may play a "bridge" role. In the follow-up experiments, we will continue to explore the effect and underlying mechanism of CD147-HE4-ANXA2 protein complex on the invasion and metastasis of ovarian cancer.

In conclusion, this study confirmed for the first time that CD147 and HE4 protein could interact with each other and were both associated with the poor prognosis of ovarian cancer, and HE4 can promote the invasion and metastasis of ovarian cancer by regulating the expression of CD147, ANXA2 may play a “bridge” role in this process, which provides a theoretical basis for the further study of underlying mechanism of CD147 and HE4 interaction in ovarian cancer.

## Supplementary Material

Supplementary figures and tables.

## Figures and Tables

**Table 1:** Expression of CD147 and HE4 in different ovarian tissues

Groups	Cases	CD147						HE4					
		-	+	++	+++	Positive (%)	High positive (%)	-	+	++	+++	Positive (%)	High positive (%)
Malignant	96	9	31	32	24	90.6 ^a,b,c^	58.3 ^d,e^	13	18	19	46	86.5 ^e,f,g^	67.7 ^h,j^
Borderline	11	4	4	2	1	63.6	27.3	5	2	2	2	54.5	36.4
Benign	14	8	4	2	0	42.9	14.3	7	3	2	2	50.0	28.6
Normal	12	8	3	1	0	33.3	8.3	8	2	1	1	33.3	16.7

**Note:** ^a,b,c^ indicated that the positive expression rate of CD147 in malignant tissues was compared with that in borderline group, benign group and normal tissues, all *P<* 0.05 (*P*_a_=0.035, *P*_b_ < 0.001, *P*_c_ < 0.001)^d,e^; indicated that the high positive rate of CD147 in malignant tissues is higher than that in benign group and normal tissues, all *P<* 0.05 (*P*_d_=0.002, *P*_e_=001); ^e,f,g^ indicated that the positive expression rate of HE4 in malignant tissues was compared with that in borderline group, benign group and normal tissues, all* P<*0.05(*P*_e_=0.024,*P*_f_ =0.003, *P*_g_ < 0.001)^h,j^; indicated that the high positive rate of HE4 in malignant tissues is higher than that in benign group and normal tissues, both *P<*0.05 (*P*_h_=0.005, *P*_j_=002).

**Table 2:** Relationships between the expression of CD147, HE4 and clinicopathological parameters of 96 ovarian cancer patients

Groups	Cases	CD147				HE4			
		Positive rate (%)	*P-*value	High expression rate (%)	*P-*value	Positive rate (%)	*P-*value	High expression rate (%)	*P-*value
**Age at diagnosis**									
<59	58	52/58 (89.7%)	*P*>0.05	33/58 (56.9%)	*P*>0.05	49/58 (84.5%)	*P*>0.05	41/58 (70.7%)	*P*>0.05
≥59	38	35/38 (92.1%)		23/38 (60.5%)		34/38 (89.5%)		24/38 (63.2%)	
**Pathological type**									
Serous	52	47/52 (90.4%)	*P*>0.05	31/52 (59.6%)	*P*>0.05	48/52 (88.5%)	*P*>0.05	35/52 (67.3%)	*P*>0.05
Mucious	7	4/7 (57.1%)		1/7 (14.3%)		4/7 (57.1%)		3/7 (42.9%)	
Endometrioid	10	9/10 (90%)		8/10 (80%)		9/10 (90%)		8/10 (80%)	
Clear cell carcinoma	8	8/8 (100%)		7/8 (87.5%)		7/8 (87.5%)		7/8 (87.5%)	
Poorly differentiated adenocarcinoma	19	19/19 (100%)		9/19 (47.4%)		17/19 (89.5%)		12/19 (63.2%)	
**FIGO stage**									
I-II	39	32/39 (82.1%)	*P*=0.043	18/39 (46.2%)	*P*=0.045	30/39 (76.9%)	*P*=0.024	19/39 (48.7%)	*P*=0.001
III-IV	57	55/57 (96.5%)		38/57 (66.7%)		53/57 (93%)		46/57 (80.7%)	
**Differentiation**									
Well	11	7/11 (63.6%)	*P*well. vs poor =0.002	2/11 (18.2%)	*P*well. vs poor =0.001	7/11 (63.6%)	*P*well. vs poor =0.030	3/11 (27.3%)	*P*well. vs poor =0.002
Moderate	28	25/28 (89.3%)		14/28 (50%)		24/28 (85.7%)		19/28 (67.9%)	
Poor	57	55/57 (96.5%)		40/57 (70.2%)		52/57 (91.2%)		43/57 (75.4%)	
**Lymphnode metastasis**									
No	57	50/57 (87.7%)	*P*>0.05	30/57 (52.6%)	*P*>0.05	47/57 (82.5%)	*P*>0.05	34/57 (59.6%)	*P*=0.039
Yes	20	19/20 (95%)		14/20 (70%)		19/20 (95%)		17/19 (85%)	
Unknown	19	18/19 (94.7%)		12/19 (63.2%)		17/19 (89.5%)		14/19 (73.7%)	

**Table 3:** The correlation between the expression of CD147 and HE4 in ovarian cancer (the Spearman correlation coefficient Rs=0.708, *P*=0.0000)

CD147	HE4		Cases
	Negative	Positive	
Negative	8	1	9
Positive	5	82	87
Cases	13	83	96

**Table 4:** The linear regression analysis of CD147 and HE4 expression in ovarian cancer

	CD147 score				HE4 score			
	univariate		multivariate		univariate		multivariate	
	β	*P*	β	*P*	β	*P*	β	*P*
HE4 score	0.342	0.000	0.31	0.001^#^				
CD147score					0.417	0.000	0.396	0.000*
Age at diagnosis	0.011	0.718			0.007	0.832		
FIGO stage	0.44	0.224			1.167	0.003		
Differentiation grade	1.203	0.014			0.997	0.068		
Lymphnode metastasis	0.773	0.381			0.489	0.618		

^#^Represents the multivariate regression analysis when CD147 score was dependent variate, including differentiation and HE4 score as independent variables; *represents the multivariate regression analysis when HE4 score was dependent variate, including FIGO stages and CD147 score as independent variables.

## Abbreviations

OC: ovarian cancer
HE4: human epididymis protein 4
WFDC2: WAP four-disulfide core protein 2
ANXA2: Annexin2
EMMPRIN: extracellular matrix metalloproteinase inducer
IHC: Immunohistochemistry
ICC: Immunocytochemistry
MMP: matrix metalloprotinase
VEGF: vascular endothelial growth factor
MAPK: mitogen-activated protein kinase
PI3K/AKT: phosphatidylinositol 3-kinase pathway/ Protein Kinase B
