# Supplementary material for: Interaction of CD147 and human epididymis protein 4 promotes invasion and metastasis of ovarian cancer
Source: J Cancer. 2021 Oct 30;12(24):7422–35. doi: 10.7150/jca.62440 (PMC8734417; doi:10.7150/jca.62440)
Supplement: Supplementary file 1 — Supplementary figures and tables. [file jcav12p7422s1.pdf]

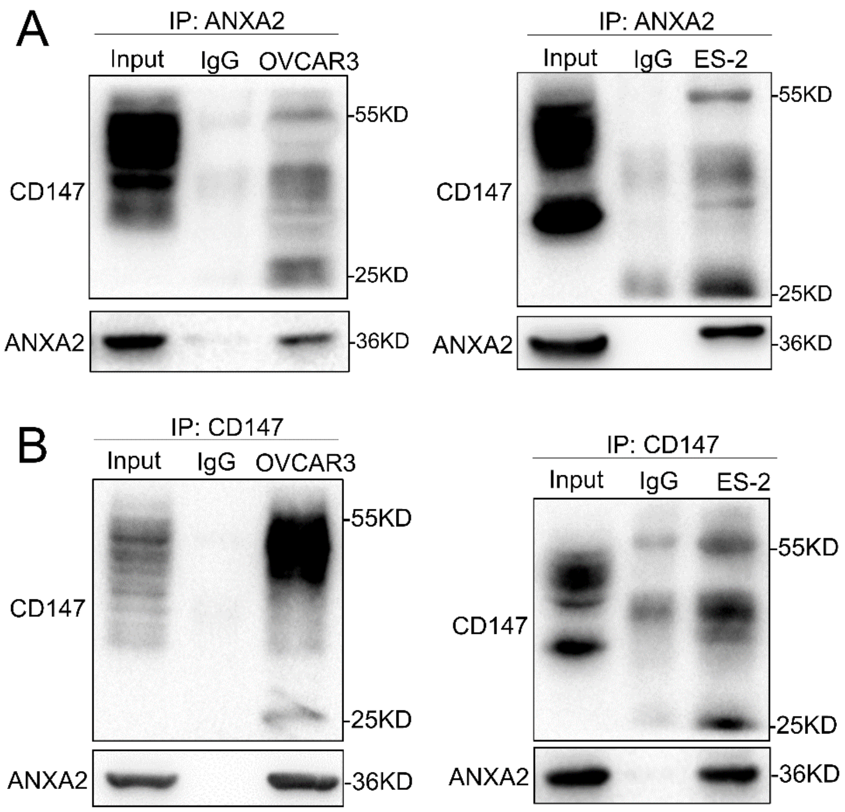

Supplementary figure 1. Co-immunoprecipitation of CD147 and ANXA2 in OVCAR3 and ES-2 cells. (A-B) Cell lysates from OVCAR3 and ES-2 cell lines were subjected to immunoprecipitation with anti-ANXA2 antibody (A) and anti-CD147 antibody (B) and then immunoblotted with anti-CD147 antibody (A) and anti-ANXA2 antibody (B), "IgG" representing the negative control. "Input" was total cell lysate of OVCAR3 and ES-2. ANXA2: annexin II

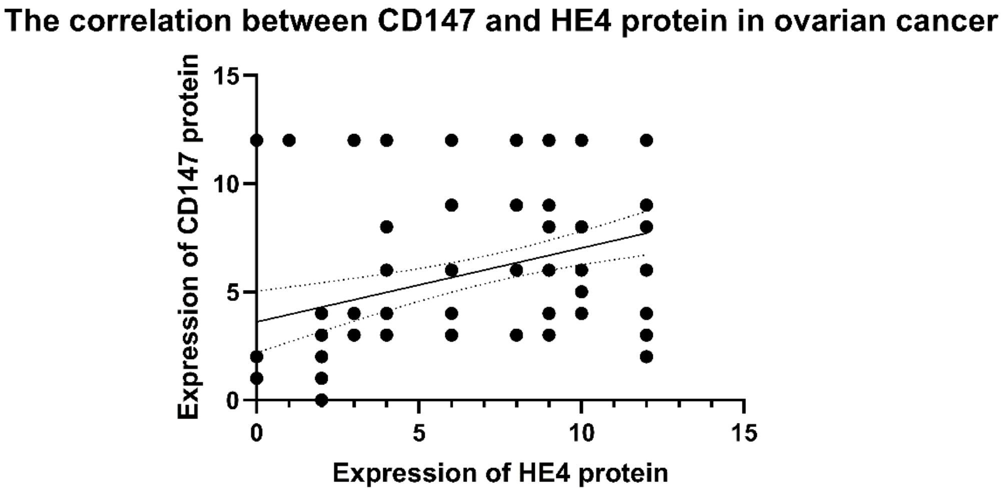

Supplementary figure 2. The correlation between the expression of CD147 and HE4 protein in ovarian cancer

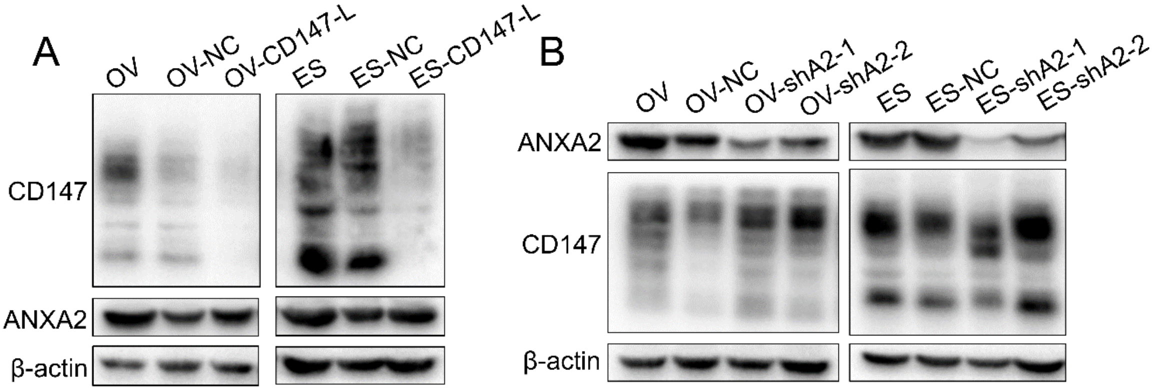

Supplementary figure 3. Interaction effects between CD147 and AXNA2 in OVCAR3 and ES-2 cells

(A) The expression of ANXA2 was not affected by the downregulation of CD147 in OVCAR3 and ES-2 cells. (B) Expression of CD147 was not affected after downregulation of ANXA2 in OVCAR3 and ES-2 cells.

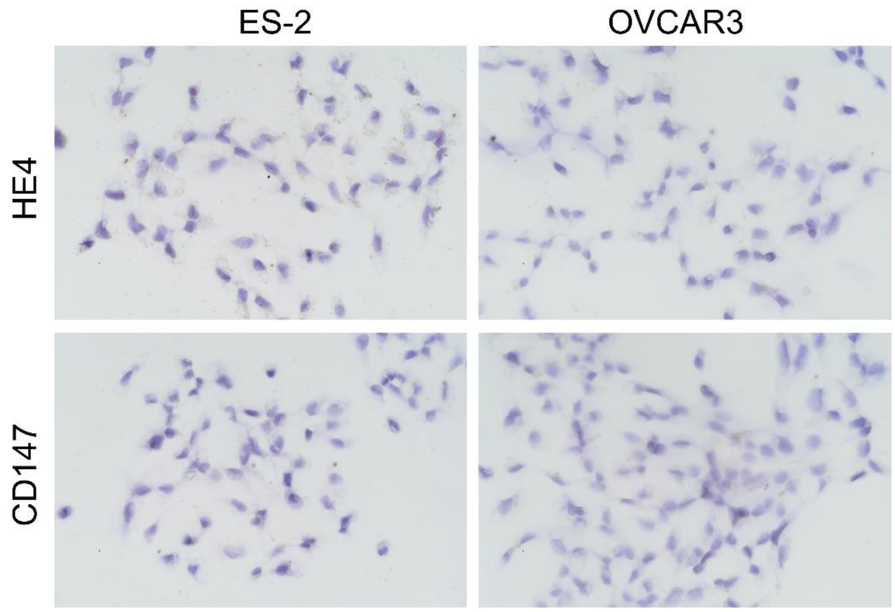

Supplementary figure 4. Secondary antibody alone served as a negative control in ovarian cancer cells by immunocytochemistry.

Supplementary Table 1 Kaplan-Meier analysis of the prognosis of ovarian cancer

Variable	Characteristics	**(Log-rank) *p*-value**
Age at diagnosis	＜59y *vs* ≥59y	**0.079**
FIGO stage	I-II *vs* III-IV	**0.001**
Differentiation grade	Well *vs* poor	**0.154**
Lymphnode metastasis	No *vs* Yes	**0.001**
CD147	Low *vs* high	**0.018**
HE4	Low *vs* high	**0.005**
